# Quantitative Amyloid Imaging Using Image-Derived Arterial Input Function

**DOI:** 10.1371/journal.pone.0122920

**Published:** 2015-04-07

**Authors:** Yi Su, Tyler M. Blazey, Abraham Z. Snyder, Marcus E. Raichle, Russ C. Hornbeck, Patricia Aldea, John C. Morris, Tammie L. S. Benzinger

**Affiliations:** 1 Department of Radiology, Washington University School of Medicine, Saint Louis, Missouri, United States of America; 2 Department of Neurology, Washington University School of Medicine, Saint Louis, Missouri, United States of America; 3 Knight Alzheimer’s Disease Research Center (ADRC), Washington University School of Medicine, Saint Louis, Missouri, United States of America; 4 Department of Neurosurgery, Washington University School of Medicine, Saint Louis, Missouri, United States of America; University of Melbourne, AUSTRALIA

## Abstract

Amyloid PET imaging is an indispensable tool widely used in the investigation, diagnosis and monitoring of Alzheimer’s disease (AD). Currently, a reference region based approach is used as the mainstream quantification technique for amyloid imaging. This approach assumes the reference region is amyloid free and has the same tracer influx and washout kinetics as the regions of interest. However, this assumption may not always be valid. The goal of this work is to evaluate an amyloid imaging quantification technique that uses arterial region of interest as the reference to avoid potential bias caused by specific binding in the reference region. 21 participants, age 58 and up, underwent Pittsburgh compound B (PiB) PET imaging and MR imaging including a time-of-flight (TOF) MR angiography (MRA) scan and a structural scan. FreeSurfer based regional analysis was performed to quantify PiB PET data. Arterial input function was estimated based on coregistered TOF MRA using a modeling based technique. Regional distribution volume (*V_T_*) was calculated using Logan graphical analysis with estimated arterial input function. Kinetic modeling was also performed using the estimated arterial input function as a way to evaluate PiB binding (*DVR_kinetic_*) without a reference region. As a comparison, Logan graphical analysis was also performed with cerebellar cortex as reference to obtain *DVR_REF_*. Excellent agreement was observed between the two distribution volume ratio measurements (r>0.89, ICC>0.80). The estimated cerebellum *V_T_* was in line with literature reported values and the variability of cerebellum *V_T_* in the control group was comparable to reported variability using arterial sampling data. This study suggests that image-based arterial input function is a viable approach to quantify amyloid imaging data, without the need of arterial sampling or a reference region. This technique can be a valuable tool for amyloid imaging, particularly in population where reference normalization may not be accurate.

## Introduction

Alzheimer’s disease (AD) is the most common form of dementia [1]. Its prevalence is expected to increase dramatically worldwide within the next 50 years as aging prevails across the globe [2]. The future success of disease-modifying therapies will depend on accurate early diagnosis of the disease before any clinical symptoms occur [3]. Although the underlying disease mechanism is still unclear, AD is characterized by two pathological hallmarks: amyloid plaques, and neurofibrillary tangles [1]. These pathological changes begin at least 10 to 20 years before clinical symptoms appear [1,4–6]. Currently, there are no disease-modifying treatments available [7], however, there is a growing consensus that effective treatment of AD may require early intervention before the onset of clinical symptoms, and well validated surrogate biomarkers are needed for the future treatment development and the design of therapy trials [8,9]. Among the various biomarkers identified, positron emission tomography (PET) imaging of the beta-amyloid (Aβ) plaques with tracers such as [^11^C]PiB [10], [^18^F]florbetapir [11], [^18^F]florbetaben [12] and [^18^F]flutemetamol[13], is the earliest indicator of AD pathology because they are capable of *in vivo* measurement of the amount of amyloid plaques in the brain. It is critical to quantify Aβ burden accurately and robustly to further our understanding of disease mechanisms, to develop early diagnostic techniques, and to identify suitable surrogate indicators for treatment monitoring and efficacy evaluation.

**Fig 1:**
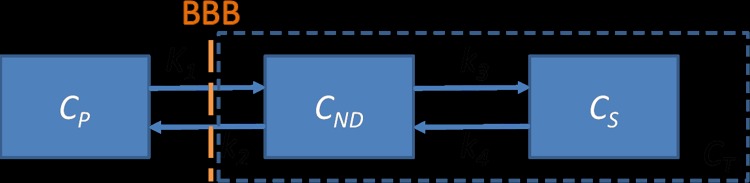
Illustration of two-tissue compartment model. Two of the compartments are located within the tissue: the nondisplaceable compartment containing free plus nonspecifically bound tracer; and the specific compartment containing specifically bound tracer. (BBB: blood-brain barrier; *C*_*P*_, tracer concentration within the vasculature compartment; *C*_*ND*_, tracer concentration within the nondisplaceable compartment; *Cs*, tracer concentration within the specific compartment; *C*_*T*_, tracer concentration in the tissue including both the nondisplaceable compartment and the specific compartment; *K*_*1*_, the influx rate constant of tracer from the vasculature compartment to the tissue; *k*_*2*_, the washout rate constant of tracer from the tissue to the vasculature; *k*_*3*_, the rate constant for tracer transfer from the nondisplaceable compartment to the specific compartment; *k*_*4*_, the rate constant for tracer transfer from specific compartment to the nondisplaceable compartment.)

With amyloid PET imaging, the amount of amyloid plaque in the brain is commonly assessed based on some variant of a two-tissue compartment kinetic model [14,15]. In this model (Fig 1), the PET tracer is assumed to present in three different forms, in the vasculature (vasculature compartment), in the brain tissue as free or nonspecifically-bound tracer (nondisplaceable compartment), and specifically bound tracer (specific compartment) [16]. The concentration of the PET tracer in these compartments are governed by a set of differential equations [17]:

$$\frac{dC_{ND}}{dt}=K_{1}C_{P}-(k_{2}+k_{3})C_{ND}+k_{4}C_{S}$$ (1)

$$\frac{dC_{S}}{dt}=k_{3}C_{ND}-k_{4}C_{S}$$ (2)

Where *C*_*P*_ is the tracer concentration within the vasculature compartment, which is also commonly referred to as the arterial input function (AIF); *C*_*ND*_ is the tracer concentration within the nondisplaceable compartment; *Cs* is the tracer concentration within the specific compartment; and the kinetic rate constants *K*_*1*_ *-k*_*4*_ describes the rate of tracer transfer among compartments (Fig 1). With PET, the tracer concentration in the tissue as a function of time (*C*_*T*_) can be directly measured. If the plasma concentration of the tracer (*C*_*P*_) is also known, then the kinetic rate constants *K*_*1*_ *-k*_*4*_ can be determined via kinetic modeling [17]. The kinetic modeling is commonly formulated as a non-linear least square fit procedure that minimizes the difference between model-based *C*_*T*_ (Eqs 3 and 4) and PET measured *C*_*T*_ [17,18].

$$C_{T}(t)=\frac{K_{1}}{\alpha_{1}-\alpha_{2}}\left[(k_{3}+k_{4}-\alpha_{1})e^{-\alpha_{1}t}+(\alpha_{2}-k_{3}-k_{4})e^{-\alpha_{2}t}\right]⊗C_{P}(t)$$ (3)

$$\alpha_{1,2}=\frac{(k_{2}+k_{3}+k_{4})\mp \sqrt{{\left(k_{2}+k_{3}+k_{4}\right)}^{2}-4k_{2}k_{4}}}{2}$$ (4)

After determination of kinetic rate constants, the binding potential (*BP*_*ND*_ = *k*_*3*_ */k*_*4*_) can be calculated [16,17], which is of specific interest in amyloid PET imaging because it is proportional to regional amyloid plaque density in the brain [16,19]. As we mentioned earlier, the kinetic modeling process requires the knowledge of *C*_*P*_. The conventional method to obtain AIF is via an invasive arterial sampling procedure [14,15]. Aside from the invasive nature, this method is also noisy and technically challenging [20,21]. To avoid arterial sampling, the current standard amyloid imaging analysis uses a reference region based approach [14,19], where, a reference region, normally cerebellar cortex, is assumed to be amyloid free and have the same tracer influx to washout ratio as the target regions of interest. Regional binding potential can be calculated by comparing region of interest PET signal against the reference region signal based on some variation of the standard two-tissue compartment model [14,19]. In one implementation of the reference region based approach, a Logan graphical analysis model with reference region (Eq 5) [22,23] is derived based on the original two-tissue compartment model.

$$\frac{\int_{0}^{T}C_{T}(t)dt}{C_{T}(T)}=DVR\left(\frac{\int_{0}^{T}C_{R}(t)dt+\frac{C_{R}(T)}{k_{2}^{'}}}{C_{T}(T)}\right)+Int$$ (5)

Where, *C*_*R*_ is the reference region tracer concentration; *k*_*2*_ *’* is the reference region washout rate constant, and DVR is the distribution volume ratio and it is related to the rate constants and binding potential as described in Eq 6, under the condition that *K*_*1*_ */k*_*2*_ = *K*_*1*_ *’*/*k*_*2*_ *’* (*K*_*1*_ *’* is the reference region influx rate constant) with no specific binding in the reference region.

$$DVR=1+\frac{k_{3}}{k_{4}}=1+BP_{ND}$$ (6)

Because of the fact that DVR is directly related to binding potential, it is also commonly used as the outcome parameter in amyloid PET imaging quantification [14,15]. Another commonly used reference region based imaging quantification measurement is the standardized uptake value ratio (SUVR) [24,25], which is simply the ratio of target region image intensity to the reference region intensity. This measurement approximates DVR with the benefits of requiring shorter scan time while introducing systematic biases [14,26]. While a cerebellar reference may be valid for many occasions, several conditions may make the assumption problematic [27], including prion disease [28,29] and familial AD [30], where amyloid deposition also occurs in the cerebellar cortex, leading to the invalidity of the assumptions of the reference region based technique. The observation of amyloid deposition in the cerebellar cortex of familial AD cases [31,32] prompted identification and validation of pons as an alternative reference region [24]. For the same reason, in a recent study from the Dominantly Inherited Alzheimer Network (DIAN), brain stem was used as the reference for quantification [4]. Nevertheless, in an earlier study that validated different amyloid imaging quantification techniques, an increase, although non-significant, in tracer uptake for pons was observed in the AD group [15]; in addition, in the study which validated pons as a reference region, an elevation in PiB retention in pons was observed in an early onset AD patient albeit at a smaller level than cerebellar cortex [24]. Therefore we cannot rule out the possibility of specific tracer uptake in the pons in patients either. Moreover, any physiological/pathological changes in the region used as reference unrelated to amyloid deposition, such as stroke, infarct, or hemorrhage, will confound the amyloid imaging quantification by altering the delivery, transport and nonspecific binding of the tracer, i.e. invalidates the assumption that *K*_*1*_ */k*_*2*_ = *K*_*1*_ *’*/*k*_*2*_ *’*.

Given the fact that reference region based techniques can be problematic in some patient populations, we decided to investigate the feasibility of quantitative analysis techniques using full kinetic modeling with AIF. As previously mentioned, the amount of amyloid deposited in the brain can be directly estimated using kinetic modeling with the knowledge of AIF without a reference region [16,17]. In fact, kinetic modeling with arterial sampling derived AIF was the gold standard technique against which the reference region based approach was validated [14,15,24]. However, conventional arterial sampling technique is invasive and challenging [20,21]. On the other hand, a number of groups have investigated the possibility of deriving AIF from imaging data without the need of arterial sampling [33–35]. Although this is an approach that has been studied for many years [34,36–40], a recent evaluation study of many of these approaches suggested none of them could reliably estimate the AIF without arterial samples [33]. We believe the key reason for the failure of these methods is the fact that they are solely dependent upon the PET data itself which does not have the spatial resolution needed to identify pure vascular signal [33,38,41–43]. Recently, we developed an imaging-derived AIF (IDAIF) technique that combines high resolution anatomical data with PET data, and demonstrated its feasibility and robustness in the context of ^15^O-water PET studies [44]. Therefore, the current goal is to adapt our IDAIF based technique for amyloid imaging to determine whether quantification can be achieved without the need of a reference region or the invasive arterial sampling procedure. We will use the IDAIF technique to quantify amyloid deposition in a cohort of normal elderly and very mild AD patients, and compare the results with conventional reference region based technique.

## Methods

### I. Participants

A total of 21 subjects were included in this study. They were all part of the Knight Alzheimer’s Disease Research Center (ADRC) research participants enrolled in longitudinal studies of memory and aging. Using a mean cortical binding potential (MCBP) cutoff of 0.18 as measured by PiB PET imaging [19], 13 were considered PiB- (MCBP<0.18), the rest (N = 8) were PiB+ (MCBP>0.18). Four out of the eight participants in the PiB+ group had a CDR score of 0.5 or greater. Demographic details are provided in Table 1. It should be pointed out that we specifically selected this population, where reference region based technique is considered valid [14,24], so that the proposed IDAIF technique can be tested against. In a future study, we will further evaluate the proposed technique in populations where the reference region based approach is problematic.

**Table 1:** Demographics for this study.

Cohort	PIB-	PIB+
**N**	13	8
**Age (SD) years**	72.5(8.6)	75.4(6.6)
**Education (SD) years**	15.4(2.1)	16.7(3.5)
**Male (%)**	6(46.2)	6(75.0)
**CDR>0 (%)**	0(0.0)	4(50)
**APOE4+ (%)**	5(38.5)	5(62.5)

#### I.1 Ethics Statement

All assessment and imaging procedures were approved by Washington University’s Human Research Protection Office, and written informed consent was obtained from all individuals or their care-givers.

### II. Imaging

PET imaging for amyloid deposition was performed using the radiotracer PiB. Preparation of PiB was carried out based on existing protocol [45]. Dynamic PET imaging was conducted with a Biograph 40 PET/CT scanner (Siemens Medical Solutions USA, Inc.) in three-dimensional mode after intravenous administration of approximately 12mCi of PiB. The images were reconstructed on a 128 x 128 x 109 matrix (2.32 x 2.32 x 2.03 mm) using filtered back-projection. Typical dynamic scans had 12 x 10-second frames, 3 x 1-minute frames, and 11 x 5-minute frames. Anatomical MRI images were acquired with T1-weighted magnetization-prepared rapid gradient echo (MPRAGE) sequence (TE = 3.16 msec, TI = 1000 msec, TR = 2400 msec) using a Siemens Trio 3T scanner with1 mm isotropic voxels. Time-of-flight (TOF) MR angiography (MRA) data was acquired (TE = 3.59 msec, TR = 23.0 msec, Flip = 18°) with 0.6 mm isotropic voxels on the same scanner.

### III. Image Analysis

The standard image analysis technique has been discussed previously [46] and a PET Unified Pipeline (PUP) (https://github.com/ysu001/PUP) has been developed by our group to facilitate automated PET data analysis. In summary, before PET data analysis using PUP, automatic brain segmentation and parcellation of the MPRAGE data was performed using FreeSurfer v5.1 (Martinos Center for Biomedical Imaging, Charlestown, Massachusetts, USA) for each participant. In all datasets, visual inspection of the automated segmentation results was performed for quality assurance purposes, and correction was done when necessary according to the FreeSurfer manual. Using PUP, raw PET images were normalized to achieve a common spatial resolution of 8mm to minimize scanner differences according to an established method [47] as a part of our standard PET processing procedure. PET to MR and TOF-MRA to MR registration were performed using a vector-gradient algorithm (VGM) [48]. Atlas registration (12-parameter affine) was performed via the MPRAGE against a standard atlas template. Inter-frame motion correction for the dynamic PET images was performed using standard image registration techniques [49]. Regional time-activity curves for each ROI were extracted by resampling the PET image to the MR space. Regional distribution volume ratio (*DVR*_*REF*_) was estimated using Logan graphical analysis with cerebellar cortex as the reference [22]. The linear fit in the Logan analysis was performed based on PET data between 30 to 60 minutes. A mean cortical *DVR* (*MCDVR*) was also calculated based on a selected set of cortical regions [46]. The washout rate constant (*k*_*2*_ *’*) of the reference region (cerebellar cortex) was set to 0.16/minute. It has previously been shown that varying *k*_*2*_ *’* over a 10-fold range (0.05 to 0.5/minute) has minimal impact on the *DVR* values [19].

### IV. IDAIF Method

**Fig 2:**
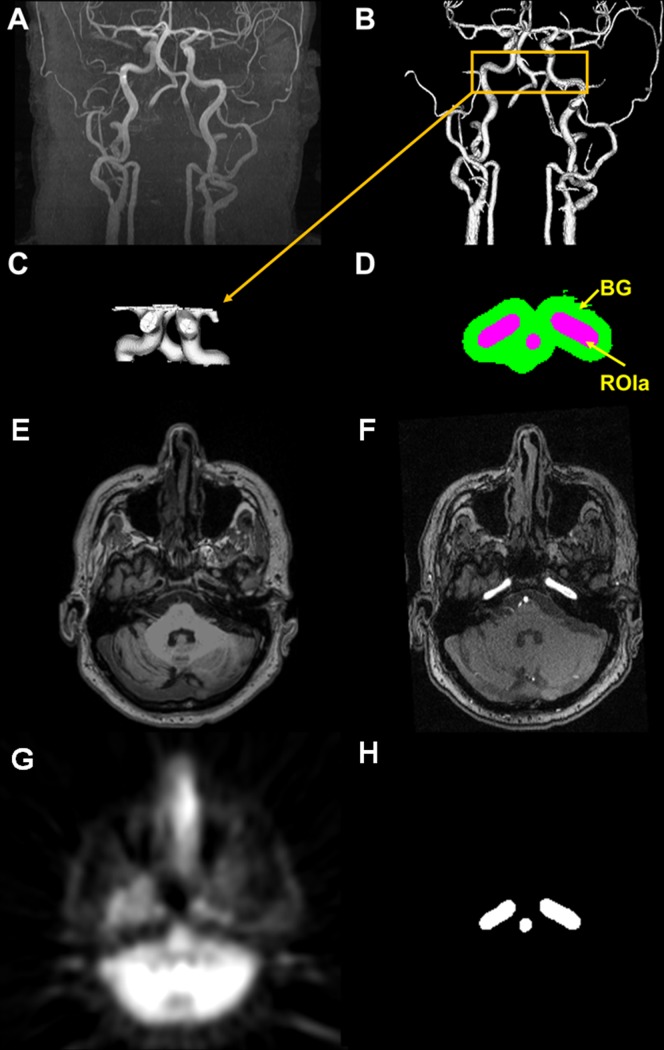
Three dimensional rendering of time-of-flight MRA (A), segmented arterial tree (B), the arterial region-of-interest (ROIa) (C), and one example slice of the arterial ROI (ROIa) and the background region-of-interest (BG) (D). Example slice of coregistered MRI (E), MRA (F), PET(G), and ROIa (H).

Similar to our previous work [44], a modified adaptive segmentation algorithm [50] is used to automatically segment the MRA images to identify arteries (Fig 2). The arterial mask is smoothed to PET resolution (8mm FWHM) and transferred to the MPRAGE space. A threshold is automatically determined to obtain a 16 cc volume after thresholding the blurred arterial mask within a rectangular box predefined in the atlas space centered at the petrous portion of the internal carotid artery. This thresholded mask is used as the arterial ROI (ROIa) for AIF estimation (Fig 2). A lower threshold is also automatically determined to obtain a 100 cc volume (ROIb) after thresholding the arterial mask, and the subtraction image of ROIa from ROIb was used as the background mask (BG) (Fig 2). The TAC curve for ROIa is assumed to follow the model below:

$$C_{{}_{ROIa}}=rC_{B}+sC_{BG}$$ (7)

The variables *r* and *s* in Eq 7 are the recovery and spill over coefficients respectively to account for the limited spatial resolution of PET; *C*_*B*_ is the whole blood activity concentration.

The AIF is derived based on fitting a kinetic model to the TAC of one large brain tissue ROI (*C*_*t*_), i.e. in our implementation the cerebellar cortex was used because it is the largest ROI generated by FreeSurfer segmentation although any large brain region can be used instead. In this study, we combined two one-tissue compartmental models [16,17] to approximate the tracer kinetics. The first one-tissue compartmental model (Eq 8) approximates the early kinetic behavior, i.e. before *C*_*t*_ peaks,

$$\frac{dC_{t}}{dt}=K_{1}C_{P}-k_{2}C_{t}$$ (8)

Where, *C*_*t*_ is tissue time activity curve in the selected ROI; *C*_*P*_ is the AIF; *K*_*1*_ and *k*_*2*_ are the influx and washout rate constant for the one-tissue compartment model. A second one-tissue compartmental model (Eq 9) approximates the two-tissue compartment kinetic behavior using a one-tissue compartment model for late time points, i.e. after 40 minutes.

$$\frac{dC_{t}}{dt}=K_{1}C_{P}-k_{2a}C_{t}$$ (9)

where, k_2a_ = k_2_/(1+k_3_/k_4_) [17]. This second one-tissue compartmental model has been proposed and used widely in the simplified reference tissue model [51]. In this model, *k*_*2a*_ is the effective washout rate constant; *k*_*2*_ is the washout rate constant for the two-tissue compartment model, and *k*_*3*_ and *k*_*4*_ are the binding rate constants. For time in between, the kinetics is described by a linear mixture of the two one-tissue compartmental models. For a given set of *C*_*t*_, *K*_*1*_, *k*_*2*_, and *k*_*2a*_, the AIF, i.e. *C*_*P*_, can be calculated as the following:

$$\{\begin{array}{c} C_{P}=\frac{1}{K_{1}}\frac{dC_{t}}{dt}+\frac{k_{2}}{K_{1}}C_{t},\;t<t_{peak}\\ C_{P}=\frac{1}{K_{1}}\frac{dC_{t}}{dt}+\frac{k_{2a}}{K_{1}}C_{t},\;t>40\;\text{min}\\ C_{P}=w_{1}\left(\frac{1}{K_{1}}\frac{dC_{t}}{dt}+\frac{k_{2}}{K_{1}}C_{t}\right)+w_{2}\left(\frac{1}{K_{1}}\frac{dC_{t}}{dt}+\frac{k_{2a}}{K_{1}}C_{t}\right),\;t_{peak}\leq t\leq 40\;\text{min} \end{array}$$ (10)

where, *w*_*1*_ and *w*_*2*_ are weighting factors, with *w*_*1*_ linearly decreases from 1 to 0 and *w*_*2*_ increases from 0 to 1.

To estimate AIF the following cost function is minimized by in a nonlinear least square fashion:

$$Q(K_{1},k_{2},K_{2a},s)=\sum_{i=1}^{F}{\left[C_{ROIa}^{PET}(i)-C_{ROIa}^{MOD}(i)\right]}^{2}$$ (11)

where, *i* is the frame index, *F* is the total number of frames, $C_{ROIa}^{PET}$ refers to PET measured TAC for ROIa, and $C_{ROIa}^{MOD}$ refers to model estimated TAC for the arterial ROI (ROIa) as modeled according to Eq 7. The recovery coefficient *r* is determined based on the arterial ROI using a 8mm FWHM Gaussian as the point spread function; *s* is estimated as one of the model parameters in Eq 11; *C*_*B*_ is estimated based on the AIF (*C*_*P*_) by applying population average parent compound ratio [14]. With two PET derived time activity curves from coregistered PET and MR (Fig 2), i.e. $C_{ROIa}^{PET}$ and *Ct*, an optimal set of parameters (*K*_*1*_, *k*_*2*_, *k*_*2a*_, and *s*) are determined that minimizes the cost *Q*, then AIF is calculated according to Eq 10.

### V. Quantification using IDAIF

With AIF determined using the method described above, for each FreeSurfer region, regional distribution volume (*V*_*T*_) is calculated using Logan analysis with arterial input [52].

$$\frac{\int_{0}^{T}C_{T}(t)dt}{C_{T}(T)}=V_{T}\frac{\int_{0}^{T}C_{P}(t)dt}{C_{T}(T)}+Int$$ (12)

$$V_{T}=\frac{K_{1}}{k_{2}}\left(1+\frac{k_{3}}{k_{4}}\right)$$ (13)

Full kinetic modeling is then performed using the IDAIF according to the two-tissue compartment model (Eqs 1–4) [17]. In this work, independent model parameters *K*_*1*_, *K*_*1*_ */k*_*2*_, and *k*_*3*_ were directly estimated using nonlinear least square fit; *V*_*T*_ is kept fixed using the value obtained from Logan graphical analysis with arterial input. Distribution volume ratio *DVR*_*kinetic*_ was then calculated as *V*_*T*_ */(K*_*1*_ */k*_*2*_ *)*.

### VI. Evaluation of the IDAIF technique

To demonstrate the validity of the IDAIF based technique for amyloid PET imaging quantification, we analyzed the quantification results in the following three aspects: 1) we compared the estimated cerebellar cortex V_T_ against literature reported values; 2) we examined whether DVR calculated using the IDAIF technique was able to differentiate the PiB+ group from PiB- group using two-tailed Welch’s t-test; 3) we compared the estimated DVR using the IDAIF technique against reference region based DVR using both Pearson’s correlation (r) and intraclass correlation (ICC) as the descriptive statistical parameters.

## Results

**Fig 3:**
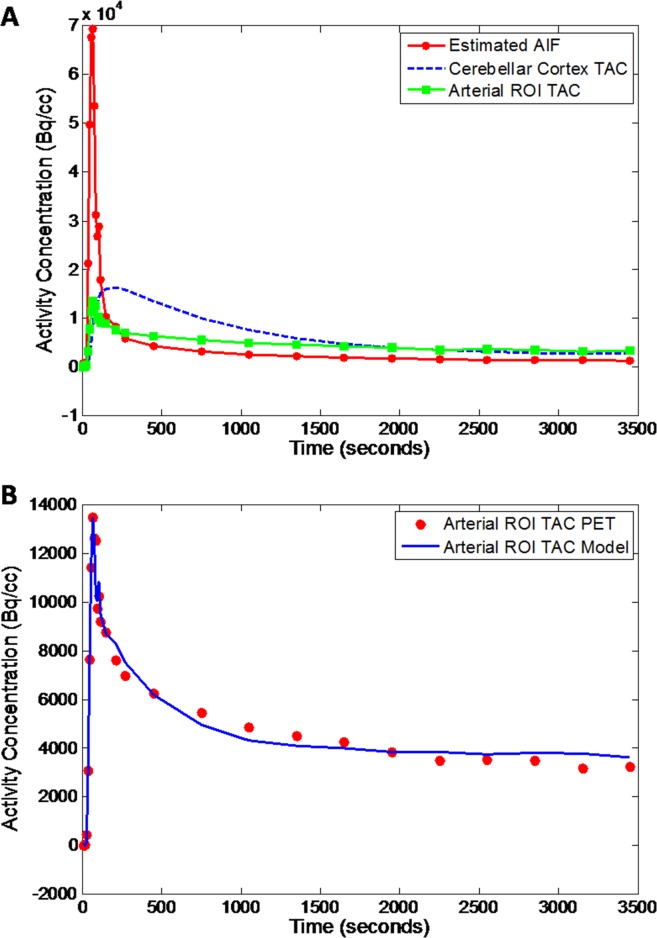
Example cerebellar cortex TAC, arterial ROI TAC and estimated AIF (A); comparison of PET measured TAC and model estimated TAC for the arterial ROI (B).

**Fig 4:**
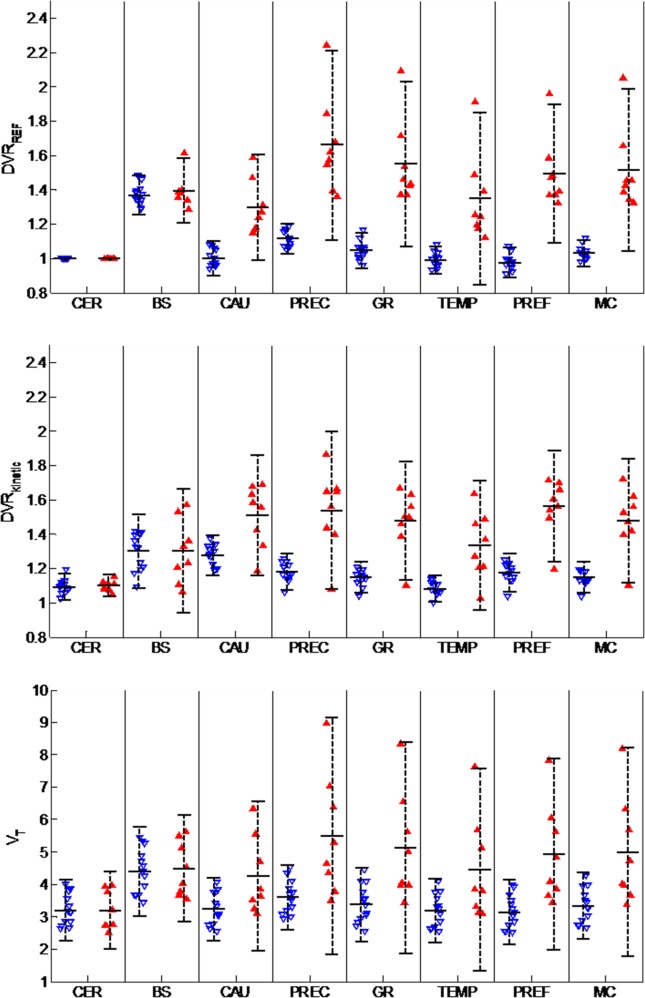
Comparison of different outcome measurements between the PiB- (open downward triangles) and the PiB+ group (solid upward triangles) for selected regions. CER: cerebellar cortex; BS: brain stem; CAU: caudate; PREC: precuneus; GR: gyrus rectus; TEMP: lateral temporal; PREF: prefrontal; MC: mean cortical. *V*_*T*_: distribution volume; *DVR*_*REF*_: target-region-to-cerebellum distribution volume ratio estimated using Logan graphical analysis with reference region (cerebellar cortex); *DVR*_*kinetic*_: *V*_*T*_ */(K1/k2)* estimated using full kinetic modeling with IDAIF.

**Fig 5:**
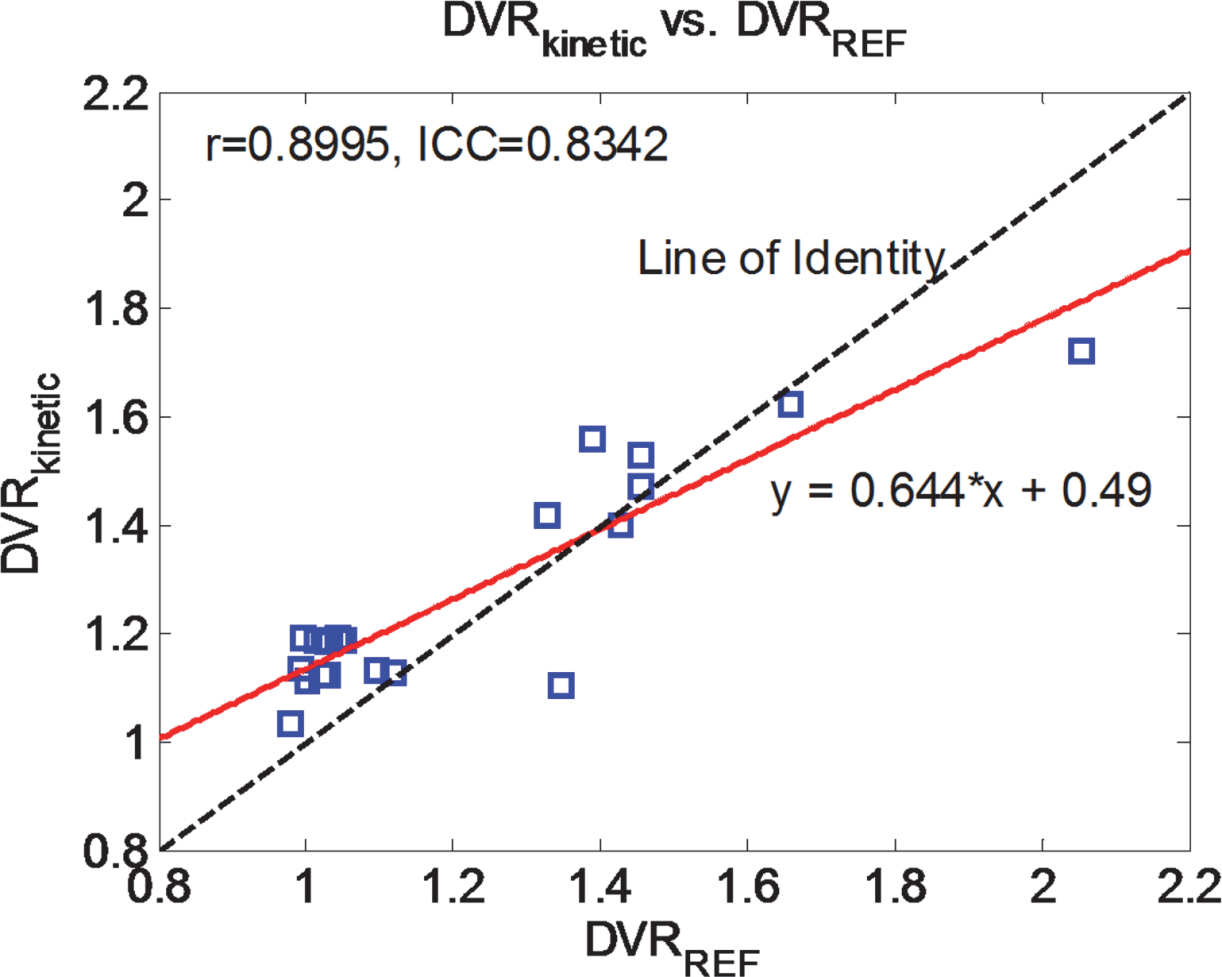
Comparisons of mean cortical DVR estimated with IDAIF and reference tissue model.

An estimated AIF using the image based technique was demonstrated in Fig 3A, and an excellent model fitting was achieved as shown in Fig 3B. Using IDAIF, estimated regional distribution volume (*V*_*T*_) was summarized in Table 2. The *V*_*T*_ for cerebellar cortex for the PiB- group was 3.20±0.47 with a 14.8% coefficient of variation. The mean value was in agreement with the reported *V*_*T*_ in control populations [15]. The variability of cerebellar *V*_*T*_ was also in line with previous studies [14,15,24]. The distribution of outcome measurements for the PiB- (N = 13) and PiB+ (N = 8) groups were illustrated in Fig 4. All the outcome measurements, i.e. the DVRs, were significantly different (p<0.01) between the two groups for caudate, precuneus, gyrus rectus, lateral temporal, prefrontal, and mean cortical regions using two-tailed Welch’s t-test. Also, as expected, the brain stem and cerebellar cortex do not show differences between the two groups. The estimated regional *DVR* by full kinetic modeling using IDAIF (*DVR*_*kinetic*_) was in excellent agreement (r = 0.8995, ICC = 0.8342) with the reference region based approach (*DVR*_*REF*_) (Fig 5). The largest discrepancies between *DVR*_*kinetic*_ (1.10) and *DVR*_*REF*_ (1.35) was 18% which was primarily due to the differences in tracer delivery and nonspecific binding between the target region (*K*_*1*_ */k*_*2*_ = 3.07 for cortical mean) and reference region (*V*_*T*_ = 2.51 for cerebellar cortex).

**Table 2:** Regional *V*_*T*_, regional *DVR* estimated using Logan analysis with cerebellar reference (*DVR*_*REF*_), and regional *DVR* estimated with full kinetic model (*DVR*_*kinetic*_).

Region	logan V_*T*_		DVR_*REF*_		DVR_*kinetic*_	
	Mean±SD (CV%)		Mean±SD (CV%)		Mean±SD (CV%)	
	PiB-	PiB+	PiB-	PiB+	PiB-	PiB+
**Cerebellar Cortex**	3.20±0.47 (14.8%)	3.21±0.61 (19.0%)	1	1	1.09±0.04 (3.7%)	1.10±0.03 (3.0%)
**Brain Stem**	4.41±0.70 (15.9%)	4.49±0.84 (18.8%)	1.37±0.06 (4.3%)	1.40±0.10 (6.9%)	1.30±0.11 (8.4%)	1.30±0.18 (14.2%)
**Precuneus**	3.60±0.51 (14.2%)*	5.50±1.86 (33.8%)*	1.12±0.05 (4.2%)*	1.66±0.28 (17.0%)*	1.18±0.05 (4.6%)*	1.54±0.23 (15.2%)*
**Temporal**	3.19±0.51 (15.9%)	4.46±1.60 (35.8%)	0.99±0.04 (4.3%)*	1.35±0.26 (19.1%)*	1.08±0.04 (3.6%)*	1.33±0.19 (14.5%)*
**Prefrontal**	3.15±0.51 (14.7%)*	4.93±1.51 (30.5%)*	0.98±0.04 (4.6%)*	1.50±0.21 (13.8%)*	1.18±0.06 (4.9%)*	1.56±0.17 (10.6%)*
**Gyrus Recus**	3.38±0.59 (17.3%)*	5.12±1.66 (32.5%)*	1.05±0.05 (5.1%)*	1.55±0.25 (15.8%)*	1.15±0.05 (4.0%)*	1.48±0.18 (11.9%)*
**Caudate**	3.23±0.50 (15.6%)*	4.25±1.17 (27.6%)*	1.00±0.05 (5.0%)*	1.30±0.16 (12.0%)*	1.28±0.06 (4.8%)*	1.51±0.18 (11.9%)*
**MC**	3.33±0.52 (15.7%)*	5.00±1.65 (32.9%)*	1.03±0.04 (3.8%)*	1.51±0.24 (15.9%)*	1.15±0.05 (4.0%)*	1.48±0.19 (12.5%)*

MC: the regions that went into the calculation of MCBP using a FreeSurfer based approach [46].

*Significantly different between PiB- and PiB+ group (p<0.05).

## Discussion

We developed and implemented a technique that estimates arterial input function from the PET images with the help of coregistered TOF-MRA data. We evaluated this technique in the context of amyloid PET imaging quantification and found excellent agreement between the AIF and reference region based quantification. The evaluation was done in a cohort of normal elderly and patients with very mild AD dementia where cerebellar cortex was a good reference region with no specific uptakes as has been shown in previous studies [14,15]. Given the linearized model for Logan graphical analysis with arterial input (Eq 12), a scaling of the AIF (*C*_*P*_) would lead to an inversely scaled *V*_*T*_. A more than 2 fold larger *V*_*T*_ was observed in Lopresti et al. [14] when uncorrected carotid ROI TAC was used as the AIF. On the other hand, our estimated *V*_*T*_ using IDAIF was in line with literature reported values. In addition, our estimated *DVR*_*kinetic*_ using a kinetic modeling approach with IDAIF for individual regions without any reference region was in good agreement with the reference region based *DVR*_*REF*_. These two facts indicated our IDAIF was an accurate representation of real plasma arterial input to the brain. We are currently pursuing a full validation study by comparing directly IDAIF based amyloid imaging quantification against arterial sampling based quantification. Nevertheless, the results presented in this study provided evidence that the IDAIF technique was a viable approach for amyloid imaging quantification without reference region.

It was observed that the variability of regional *V*_*T*_ in the PiB- group (14.8%) was higher than the *DVR* measurements (4.3% for *DVR*_*REF*_) (Table 2 and Fig 4) as observed previously [24]. Further investigation into this observation revealed a strong correlation between cerebellar cortex *V*_*T*_ and regional *K*_*1*_ */k*_*2*_ in the PiB- group (r = 0.969 for mean cortical ROI, r = 0.891 for caudate), suggesting the variation was largely due to factors that did not vary spatially. Therefore the most likely source of variability in regional *V*_*T*_ was either noise in the AIF, or variation in global perfusion, but not regional tracer delivery and transport. The fact that there is a similar level of variability in cerebellar cortex *V*_*T*_ in our study and previous studies [14,15,24] indicated that the noise level in the IDAIF was comparable to arterial sampling based AIF. This variability would be reduced by using a reference region based approach or full kinetic modeling since the effect of *K*_*1*_ and *k*_*2*_ could be removed from such analyses. There was a similar level of variability in regional *DVR* between the two methods (*DVR*_*REF*_ and *DVR*_*kinetic*_).

It should be pointed out that our proposed method used a population based parent compound ratio for metabolites correction similar to what has been done by Lopresti et al. [14] in their evaluation of image-derived AIF from carotid artery. One limitation of this approach is the potential bias caused by individual variability of tracer metabolism. This limitation can be avoided by performing metabolites analysis with venous samples, given the sparse sampling typically performed for this type of analysis [14,15]. It should also be kept in mind that parent compound ratio measurements can be noisy and may not be reliable on an individual basis.

As mentioned earlier, while using cerebellar cortex as the reference was the accepted standard for amyloid imaging quantification [14,15], in certain population such as familial AD [30–32] and prion disease [28,29], amyloid plaque has been found in the cerebellum. Because of this, Edison et al. (2012) specifically validated pons as an alternative reference. Based on results from our study, it could be seen that brain stem as defined in FreeSurfer which included the pontine region could also serve as a reference region where no detectable difference in PiB uptake between the PiB- and the PiB+ group. This supported the use of brain stem as the reference region in our previous [4,53] and ongoing studies of the DIAN cohort. Even within the group examined in this study which did not have familial AD nor prion disease, the assumption of comparable tracer delivery and nonspecific binding between the reference region and target region did not always hold. For example, when mean cortical *K*_*1*_ */k*_*2*_ was compared to cerebellum *V*_*T*_, there were three cases that had a difference greater than 15%. In the case with the largest discrepancy (22%), it led to an 18% difference between *DVR* estimated with a reference region (*DVR*_*REF*_) and a kinetic modeling approach (*DVR*_*kinetic*_) independent of reference region. Given the uncertainty in reference region validity and the effectiveness of estimating regional *DVR* using IDAIF with kinetic modeling, quantification of amyloid PET imaging using the proposed IDAIF technique would be beneficial not only in the population with suspected cerebellum uptake, but also in sporadic AD population as well.

## Conclusion

An image-based method to derive arterial input function was developed and evaluated in the context of amyloid PET imaging quantification. Excellent agreement in distribution volume measurements was observed between the IDAIF technique and the reference region based method. This demonstrates the IDAIF technique was a viable approach to quantify amyloid imaging data to eliminate the need of arterial sampling or a reference region. Further investigation is ongoing to apply this technique in population with suspected specific uptake of PiB in proposed reference tissue and to provide full validation with arterial sampling data.

## References

[1] Holtzman DM, Morris JC, Goate AM. Alzheimer's Disease: The Challenge of the Second Century. Science Translational Medicine 2011;3: 77sr71–77sr71. 10.1126/scitranslmed.3002369 21471435 PMC3130546

[2] Brookmeyer R, Johnson E, Ziegler-Graham K, Arrighi HM. Forecasting the global burden of Alzheimer's disease. Alzheimer's and Dementia 2007;3: 186–191. 10.1016/j.jalz.2007.04.381 19595937

[3] Nordberg A, Rinne JO, Kadir A, Langstrom B. The use of PET in Alzheimer disease. Nat Rev Neurol 2010;6: 78–87. 10.1038/nrneurol.2009.217 20139997

[4] Bateman RJ, Xiong C, Benzinger TL, Fagan AM, Goate A, Fox NC, et al Clinical and biomarker changes in dominantly inherited Alzheimer's disease. N Engl J Med 2012;367: 795–804. 10.1056/NEJMoa1202753 22784036 PMC3474597

[5] Jack CR Jr., Knopman DS, Jagust WJ, Shaw LM, Aisen PS, Weiner MW, et al Hypothetical model of dynamic biomarkers of the Alzheimer's pathological cascade. Lancet Neurol 2010;9: 119–128. 10.1016/S1474-4422(09)70299-6 20083042 PMC2819840

[6] Morris JC, Price AL. Pathologic correlates of nondemented aging, mild cognitive impairment, and early-stage Alzheimer's disease. JMolNeurosci 2001;17: 101–118. 10.1385/jmn:17:2:101 11816784

[7] Huang Y, Mucke L. Alzheimer mechanisms and therapeutic strategies. Cell 2012;148: 1204–1222. 10.1016/j.cell.2012.02.040 22424230 PMC3319071

[8] Aisen PS, Andrieu S, Sampaio C, Carrillo M, Khachaturian ZS, Dubois B, et al Report of the task force on designing clinical trials in early (predementia) AD. Neurology 2011;76: 280–286. 10.1212/WNL.0b013e318207b1b9 21178097 PMC3034393

[9] Aisen PS. Alzheimer's disease therapeutic research: the path forward. Alzheimers Res Ther 2009;1: 2 10.1186/alzrt2 19674435 PMC2719107

[10] Klunk WE, Engler H, Nordberg A, Wang Y, Blomqvist G, Holt DP, et al Imaging brain amyloid in Alzheimer's disease with Pittsburgh Compound-B. Ann Neurol 2004;55: 306–319. 14991808 10.1002/ana.20009

[11] Wong DF, Rosenberg PB, Zhou Y, Kumar A, Raymont V, Ravert HT, et al In vivo imaging of amyloid deposition in Alzheimer disease using the radioligand 18F-AV-45 (florbetapir [corrected] F 18). J Nucl Med 2010;51: 913–920. 10.2967/jnumed.109.069088 20501908 PMC3101877

[12] Rowe CC, Ackerman U, Browne W, Mulligan R, Pike KL, O'Keefe G, et al Imaging of amyloid beta in Alzheimer's disease with 18F-BAY94-9172, a novel PET tracer: proof of mechanism. Lancet Neurol 2008;7: 129–135. 10.1016/S1474-4422(08)70001-2 18191617

[13] Vandenberghe R, Van Laere K, Ivanoiu A, Salmon E, Bastin C, Triau E, et al 18F-flutemetamol amyloid imaging in Alzheimer disease and mild cognitive impairment: a phase 2 trial. Ann Neurol 68: 319–329. 10.1002/ana.22068 20687209

[14] Lopresti BJ, Klunk WE, Mathis CA, Hoge JA, Ziolko SK, Lu X, et al Simplified quantification of Pittsburgh Compound B amyloid imaging PET studies: a comparative analysis. J Nucl Med 2005;46: 1959–1972. 16330558

[15] Price JC, Klunk WE, Lopresti BJ, Lu X, Hoge JA, Ziolko SK, et al Kinetic modeling of amyloid binding in humans using PET imaging and Pittsburgh Compound-B. J Cereb Blood Flow Metab 2005;25: 1528–1547. 15944649 10.1038/sj.jcbfm.9600146

[16] Innis RB, Cunningham VJ, Delforge J, Fujita M, Gjedde A, Gunn RN, et al Consensus nomenclature for in vivo imaging of reversibly binding radioligands. J Cereb Blood Flow Metab 2007;27: 1533–1539. 17519979 10.1038/sj.jcbfm.9600493

[17] Koeppe RA, Holthoff VA, Frey KA, Kilbourn MR, Kuhl DE. Compartmental analysis of [11C]flumazenil kinetics for the estimation of ligand transport rate and receptor distribution using positron emission tomography. J Cereb Blood Flow Metab 1991;11: 735–744. 1651944 10.1038/jcbfm.1991.130

[18] Su Y, Shoghi KI. Wavelet denoising in voxel-based parametric estimation of small animal PET images: a systematic evaluation of spatial constraints and noise reduction algorithms. Phys Med Biol 2008;53: 5899–5915. 10.1088/0031-9155/53/21/001 18836221 PMC4283464

[19] Mintun MA, Larossa GN, Sheline YI, Dence CS, Lee SY, Mach RH, et al [11C]PIB in a nondemented population: potential antecedent marker of Alzheimer disease. Neurology 2006;67: 446–452. 16894106 10.1212/01.wnl.0000228230.26044.a4

[20] Vaishnavi SN, Vlassenko AG, Rundle MM, Snyder AZ, Mintun MA, Raichle ME. Regional aerobic glycolysis in the human brain. Proc Natl Acad Sci U S A 2010;107: 17757–17762. 10.1073/pnas.1010459107 20837536 PMC2955101

[21] Derdeyn CP, Videen TO, Simmons NR, Yundt KD, Fritsch SM, Grubb RL Jr., et al Count-based PET method for predicting ischemic stroke in patients with symptomatic carotid arterial occlusion. Radiology 1999;212: 499–506. 10429709 10.1148/radiology.212.2.r99au27499

[22] Logan J, Fowler JS, Volkow ND, Wang GJ, Ding YS, Alexoff DL. Distribution volume ratios without blood sampling from graphical analysis of PET data. Journal of Cerebral Blood Flow and Metabolism 1996;16: 834–840. 8784228 10.1097/00004647-199609000-00008

[23] Logan J. Graphical analysis of PET data applied to reversible and irreversible tracers. Nucl Med Biol 2000;27: 661–670. 11091109 10.1016/s0969-8051(00)00137-2

[24] Edison P, Hinz R, Ramlackhansingh A, Thomas J, Gelosa G, Archer HA, et al Can target-to-pons ratio be used as a reliable method for the analysis of [(11)C]PIB brain scans? Neuroimage 2012;60: 1716–1723. 10.1016/j.neuroimage.2012.01.099 22306804

[25] Lowe VJ, Kemp BJ, Jack CR Jr., Senjem M, Weigand S, Shiung M, et al Comparison of 18F-FDG and PiB PET in cognitive impairment. J Nucl Med 2009;50: 878–886. 10.2967/jnumed.108.058529 19443597 PMC2886669

[26] Koeppe RA, Frey KA, Kume A, Albin R, Kilbourn MR, Kuhl DE. Equilibrium versus compartmental analysis for assessment of the vesicular monoamine transporter using (+)-alpha-[11C]dihydrotetrabenazine (DTBZ) and positron emission tomography. J Cereb Blood Flow Metab 1997;17: 919–931. 9307605 10.1097/00004647-199709000-00001

[27] Edison P, Brooks DJ, Turkheimer FE, Archer HA, Hinz R. Strategies for the generation of parametric images of [11C]PIB with plasma input functions considering discriminations and reproducibility. Neuroimage 2009;48: 329–338. 10.1016/j.neuroimage.2009.06.079 19591948

[28] Mead S, Poulter M, Beck J, Webb TE, Campbell TA, Linehan JM, et al Inherited prion disease with six octapeptide repeat insertional mutation—molecular analysis of phenotypic heterogeneity. Brain 2006;129: 2297–2317. 16923955 10.1093/brain/awl226

[29] Watanabe R, Duchen LW. Cerebral amyloid in human prion disease. Neuropathol Appl Neurobiol 1993;19: 253–260. 8355811 10.1111/j.1365-2990.1993.tb00435.x

[30] Kaufer DI, Yuen H, DeKosky ST, Klunk WE. P1-256: Cerebellar amyloid in a case of early-onset dementia with a presenilin-1 mutation: Correlation to clinical phenotype. Alzheimer's & dementia: the journal of the Alzheimer's Association 2008;4: T290–T291.

[31] Lippa CF, Saunders AM, Smith TW, Swearer JM, Drachman DA, Ghetti B, et al Familial and sporadic Alzheimer's disease: neuropathology cannot exclude a final common pathway. Neurology 1996;46: 406–412. 8614503 10.1212/wnl.46.2.406

[32] Mann DM, Pickering-Brown SM, Takeuchi A, Iwatsubo T. Amyloid angiopathy and variability in amyloid beta deposition is determined by mutation position in presenilin-1-linked Alzheimer's disease. Am J Pathol 2001;158: 2165–2175. 11395394 10.1016/s0002-9440(10)64688-3 PMC1891993

[33] Zanotti-Fregonara P, Fadaili EM, Maroy R, Comtat C, Souloumiac A, Jan S, et al Comparison of eight methods for the estimation of the image-derived input function in dynamic [lsqb]18F[rsqb]-FDG PET human brain studies. J Cereb Blood Flow Metab 2009;29: 1825–1835. 10.1038/jcbfm.2009.93 19584890

[34] Fang YH, Muzic RF Jr. Spillover and Partial-Volume Correction for Image-Derived Input Functions for Small-Animal 18F-FDG PET Studies. J Nucl Med 2008;49: 606–614. 10.2967/jnumed.107.047613 18344438

[35] Su Y, Welch MJ, Shoghi KI. Single input multiple output (SIMO) optimization for input function estimation: a simulation study. Nuclear Science Symposium Conference Record, 2007 NSS '07 IEEE. 2007. pp. 4481–4484.

[36] Croteau E, Lavallée É, Labbe S, Hubert L, Pifferi F, Rousseau J, et al. Image-derived input function in dynamic human PET/CT: methodology and validation with 11C-acetate and 18F-fluorothioheptadecanoic acid in muscle and 18F-fluorodeoxyglucose in brain. European Journal of Nuclear Medicine and Molecular Imaging 2010. 10.1007/s00259-010-1443-z PMC2914861 20437239

[37] Mourik JEM, Lubberink M, Klumpers UMH, Comans EF, Lammertsma AA, Boellaard R. Partial volume corrected image derived input functions for dynamic PET brain studies: Methodology and validation for [11C]flumazenil. Neuroimage 2008;39: 1041–1050. 18042494 10.1016/j.neuroimage.2007.10.022

[38] Sitek A, Gullberg GT, Huesman RH. Correction for ambiguous solutions in factor analysis using a penalized least squares objective. IEEE Trans Med Imaging 2002;21: 216–225. 11989846 10.1109/42.996340

[39] Feng D, Wong KP, Wu CM, Siu WC. A technique for extracting physiological parameters and the required input function simultaneously from PET image measurements: theory and simulation study. IEEE Trans Inf Technol Biomed 1997;1: 243–254. 11020827 10.1109/4233.681168

[40] Houston AS. The effect of apex-finding errors on factor images obtained from factor analysis and oblique transformation. Phys Med Biol 1984;29: 1109–1116. 6483975 10.1088/0031-9155/29/9/007

[41] Su Y, Welch MJ, Shoghi KI. The application of maximum likelihood factor analysis (MLFA) with uniqueness constraints on dynamic cardiac microPET data. Phys Med Biol 2007;52: 2313–2334. 17404471 10.1088/0031-9155/52/8/018

[42] El Fakhri G, Sitek A, Guerin B, Kijewski MF, Di Carli MF, Moore SC. Quantitative dynamic cardiac 82Rb PET using generalized factor and compartment analyses. J Nucl Med 2005;46: 1264–1271. 16085581

[43] Zanotti-Fregonara P, Maroy R, Comtat C, Jan S, Gaura V, Bar-Hen A, et al Comparison of 3 methods of automated internal carotid segmentation in human brain PET studies: application to the estimation of arterial input function. J Nucl Med 2009;50: 461–467. 10.2967/jnumed.108.059642 19223421

[44] Su Y, Arbelaez AM, Benzinger TL, Snyder AZ, Vlassenko AG, Mintun MA, et al Noninvasive estimation of the arterial input function in positron emission tomography imaging of cerebral blood flow. J Cereb Blood Flow Metab 2013;33: 115–121. 10.1038/jcbfm.2012.143 23072748 PMC3597366

[45] Mathis CA, Wang Y, Holt DP, Huang GF, Debnath ML, Klunk WE. Synthesis and evaluation of 11C-labeled 6-substituted 2-arylbenzothiazoles as amyloid imaging agents. JMedChem 2003;46: 2740–2754. 12801237 10.1021/jm030026b

[46] Su Y, D'Angelo GM, Vlassenko AG, Zhou G, Snyder AZ, Marcus DS, et al Quantitative analysis of PiB-PET with FreeSurfer ROIs. PLoS One 2013;8: e73377 10.1371/journal.pone.0073377 24223109 PMC3819320

[47] Joshi A, Koeppe RA, Fessler JA. Reducing between scanner differences in multi-center PET studies. Neuroimage 2009;46: 154–159. 10.1016/j.neuroimage.2009.01.057 19457369 PMC4308413

[48] Rowland DJ, Garbow JR, Laforest R, Snyder AZ. Registration of [18F]FDG microPET and small-animal MRI. Nucl Med Biol 2005;32: 567–572. 16026703 10.1016/j.nucmedbio.2005.05.002

[49] Hajnal JV, Saeed N, Soar EJ, Oatridge A, Young IR, Bydder GM. A registration and interpolation procedure for subvoxel matching of serially acquired MR images. J Comput Assist Tomogr 1995;19: 289–296. 7890857 10.1097/00004728-199503000-00022

[50] Wilson DL, Noble JA. An adaptive segmentation algorithm for time-of-flight MRA data. IEEE Trans Med Imaging 1999;18: 938–945. 10628953 10.1109/42.811277

[51] Lammertsma AA, Hume SP. Simplified reference tissue model for PET receptor studies. Neuroimage 1996;4: 153–158. 9345505 10.1006/nimg.1996.0066

[52] Logan J, Fowler JS, Volkow ND, Wolf AP, Dewey SL, Schlyer DJ, et al Graphical analysis of reversible radioligand binding from time-activity measurements applied to [N-11C-methyl]-(-)-cocaine PET studies in human subjects. J Cereb Blood Flow Metab 1990;10: 740–747. 2384545 10.1038/jcbfm.1990.127

[53] Benzinger TL, Blazey T, Jack CR Jr., Koeppe RA, Su Y, Xiong C, et al. Regional variability of imaging biomarkers in autosomal dominant Alzheimer's disease. Proc Natl Acad Sci U S A 2014. 10.1073/pnas.1317918110 PMC3839740 24194552

